# The placement of chest tubes by emergency physicians might diminish the time from diagnosis to treatment in patients with pneumothorax

**DOI:** 10.1186/1757-7241-20-S2-P48

**Published:** 2012-04-16

**Authors:** David Levarett Buck, Thomas Andersen Schmidt

**Affiliations:** 1 The Emergency Department, Holbæk Hospital, Holbæk, Denmark

## Background

In Denmark the placement of chest tubes is routinely performed by the surgical specialties, namely thoracic, orthopaedic and abdominal surgeons. At our emergency department (ED) we have trained emergency physicians in the placement of chest tubes in patients presenting with a pneumothorax. We postulate that patient discomfort associated with dyspnoea from a pneumothorax can be mitigated by shortening the time from diagnosis to definitive therapy. Our study is retrospective and observational in design, and evaluates the placement of chest tubes by an emergency physician in the ED when compared to a surgeon performing the same procedure in the operating room (OR).

## Methods

All adult patients admitted to the hospital from our ED between 1-JAN-2010 to 31-APR-2011 with the diagnosis of pneumothorax were retrieved.

The time from pneumothorax confirmed by X-ray to placement of chest tube (ΔtDrain) was calculated. The two groups were compared using two tailed unpaired student’s t-test.

## Results

Thirty patients were admitted to the hospital from our ED with the diagnosis of pneumothorax. Of these 30 patients, 13 were excluded: five were misdiagnosed, four were observed without treatment, two had delayed insertion of chest tubes following progression of the pneumothorax, and two had chest tubes inserted at the ED with the assistance of surgeons.

Of the 17 patients included, 12 had chest tubes inserted by surgeons in the OR, and 5 by emergency physicians at the ED. No complications were described. The mean ΔtDrain was 4:01 hours (range 00:46-9:31) when chest tubes were inserted by surgeons in the OR, which was significantly longer than ΔtDrain of 1:54 hours (range 0:45-3:20) when inserted by emergency physicians at the ED (p=0.04).

## Conclusion

This study is limited by the few numbers of patients studied, its retrospective design, and is unable to detect possible differences in the frequency of complications between the two study groups. Nevertheless it clearly indicates that ΔtDrain might be diminished substantially when chest tubes are inserted by emergency physicians at the ED. We therefore suggest that insertion of chest tubes should be mastered by emergency physicians, and that it should be a standard procedure at the ED.

